# Analysis of *nifH* DNA and RNA reveals a disproportionate contribution to nitrogenase activities by rare plankton-associated diazotrophs

**DOI:** 10.1186/s12866-019-1565-9

**Published:** 2019-08-15

**Authors:** Qing-Song Yang, Jun-De Dong, Manzoor Ahmad, Juan Ling, Wei-Guo Zhou, Ye-Hui Tan, Yuan-Zhou Zhang, Dan-Dan Shen, Yan-Ying Zhang

**Affiliations:** 1 CAS Key Laboratory of Tropical Marine Bio-resources and Ecology, Guangdong Provincial Key Laboratory of Applied Marine Biology, South China Sea Institute of Oceanology, Chinese Academy of Sciences, Guangzhou, 510301 China; 2 Tropical Marine Biological Research Station in Hainan, South China Sea Institute of Oceanology, Chinese Academy of Sciences, Sanya, 572000 China; 3 University of Chinese Academy of Sciences, Beijing, 100049 China; 4 State Oceanic Administration Sansha Marine Environmental Monitoring Center Station, Haikou, 570311 China; 5 Section of Biological Oceanography, Leibniz Institute for Baltic Sea Research, 18119 Warnemünde, Germany

**Keywords:** Symbiosis, Nitrogen fixation, Presence-absence, Transcriptional activity, Dormancy, South China Sea

## Abstract

**Background:**

Holobionts comprising nitrogen-fixing diazotrophs and phytoplankton or zooplankton are ubiquitous in the pelagic sea. However, neither the community structure of plankton-associated diazotrophs (PADs) nor their nitrogenase transcriptional activity are well-understood. In this study, we used *nifH* gene Illumina sequencing and quantitative PCR to characterize the community composition and *nifH* expression profile of PADs with > 100 μm size fraction in the euphotic zone of the northern South China Sea.

**Results:**

The results of DNA- and RNA-derived *nifH* gene revealed a higher alpha-diversity in the active than in the total community. Moreover, the compositional resemblance among different sites was less for active than for total communities of PADs. We characterized the 20 most abundant OTUs by ranking the sum of sequence reads across 9 sampling stations for individual OTUs in both *nifH* DNA and RNA libraries, and then assessed their phylogenetic relatedness. Eight of the 20 abundant OTUs were phylogenetically affiliated with *Trichodesmium* and occurred in approximately equal proportion in both the DNA and RNA libraries. The analysis of *nifH* gene expression level showed uneven attribute of the abundance and nitrogenase activities by the remaining 12 OTUs. Taxa belonging to cluster III and *Betaproteobacteria* were present at moderate abundance but exhibited negligible nitrogenase transcription activity. Whereas, the abundances of *Richelia*, *Deltaproteobacteria* and *Gammaproteobacteria* were low but the contribution of these groups to nitrogenase transcription was disproportionately high.

**Conclusions:**

The substantial variation in community structure among active dizatrophic fractions compared to the total communities suggests that the former are better indicators of biological response to environmental changes. Altogether, our study highlights the importance of rare PADs groups in nitrogen fixation in plankton holobionts, evidenced by their high level of nitrogenase transcription.

**Electronic supplementary material:**

The online version of this article (10.1186/s12866-019-1565-9) contains supplementary material, which is available to authorized users.

## Background

Nitrogen fixation by marine diazotrophs compensates for the loss of nitrogen through sinking and denitrification [1] and supports primary productivity in marine ecosystems [2–4]. The dominant N_2_ fixers in the world’s oceans are diazotrophic cyanobacteria, which are composed of three major groups: filamentous non-heterocyst-forming cyanobacteria, heterocystous cyanobacteria, and unicellular cyanobacteria [5]. *Trichodesmium*, a genus of filamentous non-heterocystous cyanobacteria, is thought to be the major contributor to marine nitrogen fixation globally [6–8]. As members of heterocystous cyanobacteria, the *Richelia* co-exist with diatoms and are commonly present in the open oceans [9, 10]. A recent investigation of a unicellular cyanobacteria group (UCYN-A) pointed out the need to re-evaluate the underestimated contribution of unicellular cyanobacteria to marine nitrogen fixation [11]. In recent years, the *nifH* gene, a gene encoding a subunit of nitrogenase enzyme, has been frequently used to explore the diversity of diazotroph [12]. In addition to autotrophic diazotrophic cyanobacteria, the *nifH* gene maker have identified highly diversified non-cyanobacterial heterotrophic diazotrophs in a wide oligotrophic aquatic environments [12, 13].

Holobionts comprising nitrogen-fixing diazotrophs and phytoplankton or zooplankton are ubiquitous in the pelagic sea [14, 15]. *Trichodesmium* in the holobionts form large colonies (millimeter size), and provide a micro-niche for diverse assemblages of microorganisms including autotrophic and heterotrophic diazotrophs. Despite that *Trichodesmium* is non-symbiotic diazotrophs, its colony-forming lifestyle sustains the growth of diverse symbiotic diazotrophic species [16, 17]. Various symbiotic strategies between plankton-associated diazotrophs (PADs) and their hosts facilitate marine biologic nitrogen fixation in the holobionts. Symbionts of diazotrophic cyanobacteria are exemplified by the genus *Richelia,* displaying symbiotic association with eukaryotic algae *Rhizosolenia* and other diatoms [9, 18–21]. The symbiotic strategies of autotrophic cyanobacterial diazotrophs, including symbiotic unicellular cyanobacteria [22], have been extensively documented. Nonetheless, the symbiotic phenomenon is not unique to autotrophic cyanobacterial diazotrophs and also exists in heterotrophic nitrogen-fixing bacteria [23–25]. This was demonstrated in a ^15^N-incorporation experiment that showed N_2_ fixation in the copepod gut by heterotrophic symbiotic diazotrophs, and the vital role of that symbiosis in marine nitrogen cycles [26].

The diversity and distribution of PADs have been extensively studied by means of DNA-derived *nifH* clone libraries [13, 24–26] and high-throughput sequencing [27]. However, due to the high degree of dormancy of environmental microbes [28, 29], the contribution of nitrogen fixation and community dynamics may be incorrectly estimated if based solely on data obtained from *nifH* DNA analysis [11, 30]. Instead, the use of both DNA- and RNA-derived assessments of microbial communities allows the additional identification of the rare but metabolically active microbes present in natural environments [31–33]. In the case of PADs, despite their key role in converting nitrogen in marine ecosystems, their nitrogenase transcription activity is poorly understood. Moreover, differences in the abundance and activity of different PADs groups have yet to be investigated.

Non-cyanobacteria predominated the bulk diazotrophic communities of the northern South China Sea in previous field studies [34, 35] whereas the community structure of the PADs remains largely unexplored. Thus, in the present study, we extended previous work and used high-throughput *nifH* gene amplicon sequencing to characterize the total and active communities of PADs inhabiting the northern South China Sea. In addition, by analyzing *nifH* gene expression, we were able to identify differences in nitrogenase transcription activity by the different phylogenetic groups of PADs depending on their symbiotic strategies.

## Results

### Alpha- and beta-diversity

We retrieved a total of 762,968 valid *nifH* sequences from the 18 DNA- and RNA-derived libraries. After quality filtering, the valid sequences (310–380 bp) were clustered into 731 OTUs at the 94% sequence similarity level. From a total of 731 OTUs, 153 OTUs were present in all nine samples of the DNA library, 109 OTUs in all nine samples of the RNA library, and 104 OTUs in both the DNA and the RNA libraries. For simplicity, we refer to *nifH* DNA-sequence composition as ‘total community’, while *nifH* RNA-sequence composition as ‘active community’ throughout the manuscript.

**Fig. 1:**
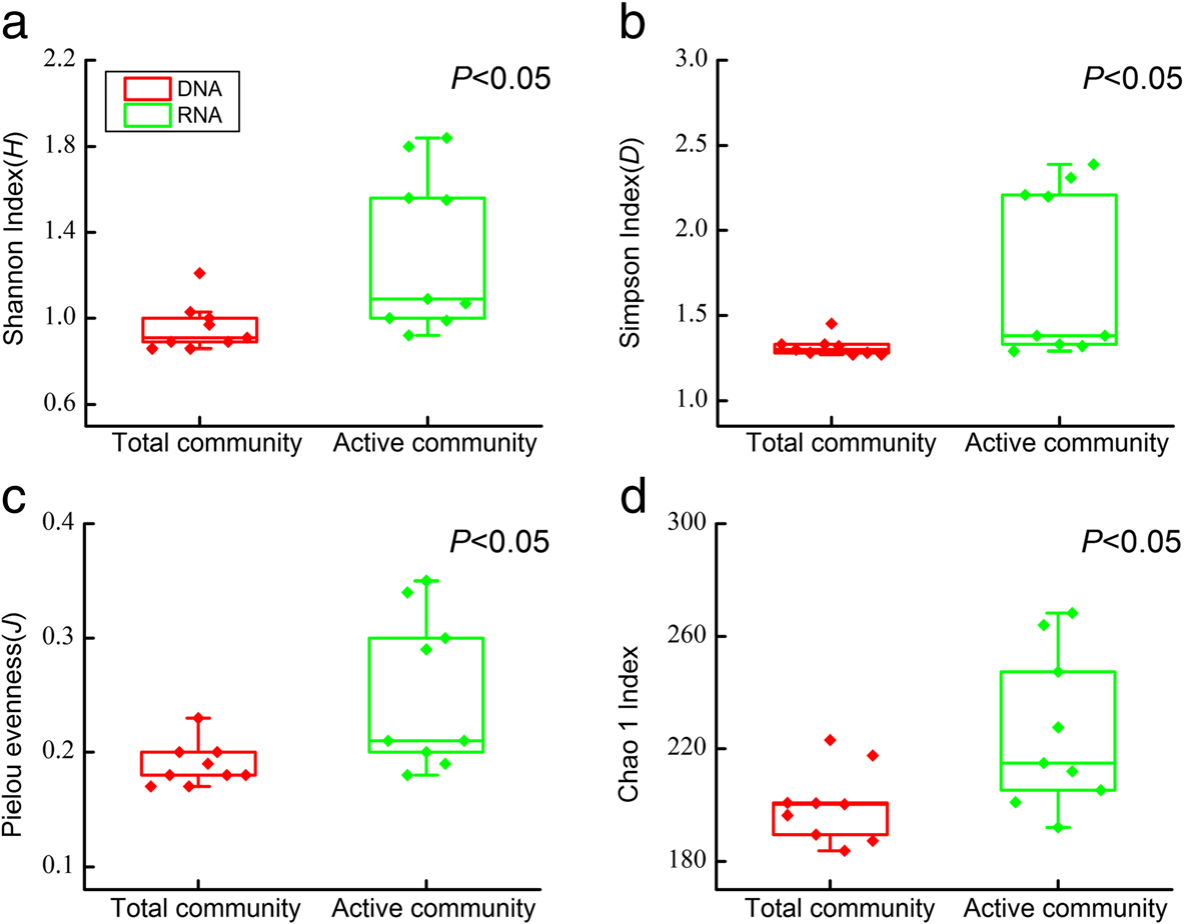
Alpha-diversity of the total and active plankton-associated diazotrophs (PADs) communities. Shannon index (**a**), Simpson index (**b**), Pielou evenness (**c**) and Chao 1 index (**d**) of the total and active PADs community based on *nifH*-derived DNA and RNA libraries. The dots indicate the diversity index value of each sample

To evaluate the diversity of the diazotrophic assemblages in the total and active PADs communities, the Shannon index, Simpson index, Pielou evenness, and Chao 1 index, as alpha-diversity parameters, were determined in each sample based on the number of *nifH*-sequence-derived OTUs. Generally, each alpha-diversity parameter was significantly higher in the active than in the total communities (ANOVA, *P* < 0.05; Fig. 1), despite some variation in each diversity index among the stations (Additional file 1: Table S2).

**Fig. 2:**
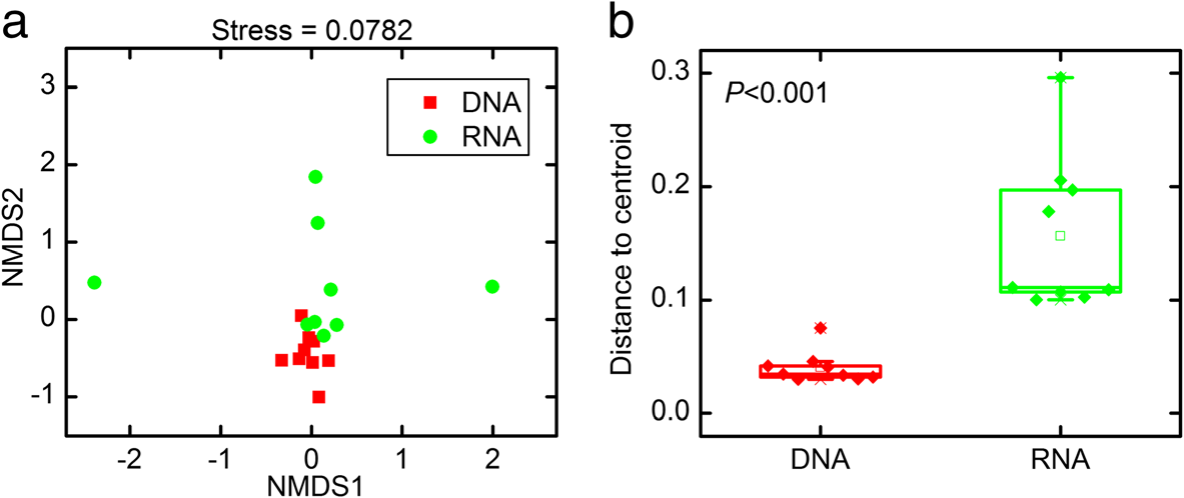
Beta-diversity of the total and active PADs communities. **a** Similarity among total and active PADs communities as determined by non-metric multidimensional scaling ordination. **b** Beta-dispersion illustrating the difference in the between-community variation of the *nifH* DNA and *nifH* RNA libraries. The significance of the between-community variation within total and active PADs was statistically tested by ANOVA

The beta-diversity of the total and active PADs communities was investigated as well, with the differences in between-group community composition visualized in a NMDS plot (Fig. 2a). The total PADs communities were taxonomically distinct according to their sampling origin (average Bray-Curtis distance, 0.06 ± 0.02). However, regardless of their origin, the total PADs communities clustered together in the NMDS scatter plot whereas the active PADs communities were more dispersed (average Bray-Curtis distance, 0.22 ± 0.11; Fig. 2a). To better understand the mean between-community differences in dependence of presence-absence or metabolic activity, a beta-dispersion analysis was performed to quantify the between-group variation within the total and active PADs communities. Between-community variation was significantly lower in the total PADs communities than in the active fractions (ANOVA, *P* < 0.001; Fig. 2b), suggesting that the differences in the diazotrophic communities across all stations with respect to the presence-absence of the *nifH* gene were smaller than the differences in *nifH* expression.

### The composition and environmental relationships of diazotrophs

**Fig. 3:**
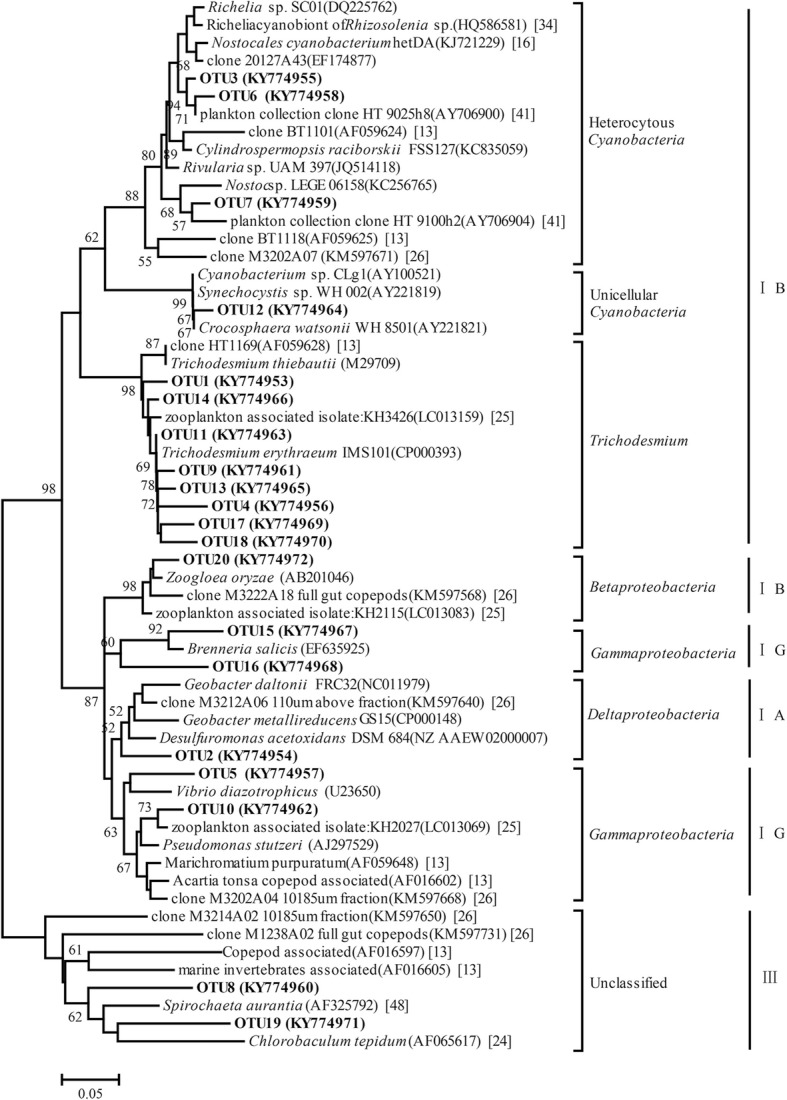
Unrooted neighbor-joining phylogenetic tree based on the *nifH* amino acid sequences of the 20 most abundant OTUs and reference sequences. The bootstrap values (> 50%) of relevant nodes are shown based on 1000 replicates. Sequences from this study are shown in bold. Scale bars: 5% of the estimated sequence divergence. The number in square brackets refers to the studies in which the reference sequences were previously reported

To visualize the phylogenetic diversity of the diazotrophs, a phylogenetic tree was constructed using the *nifH* sequences of the 20 most abundant OTUs (comprising 90.3% of the total *nifH* DNA and RNA sequences) and reference sequences (Fig. 3). The retrieved *nifH* sequences belonged to a wide range of diazotrophic groups, including *Trichodesmium*, heterocystous cyanobacteria, unicellular cyanobacteria, *Gammaproteobacteria*, *Deltaproteobacteria*, *Betaproteobacteria* and diazotrophs of cluster III (Fig. 3).

**Fig. 4:**
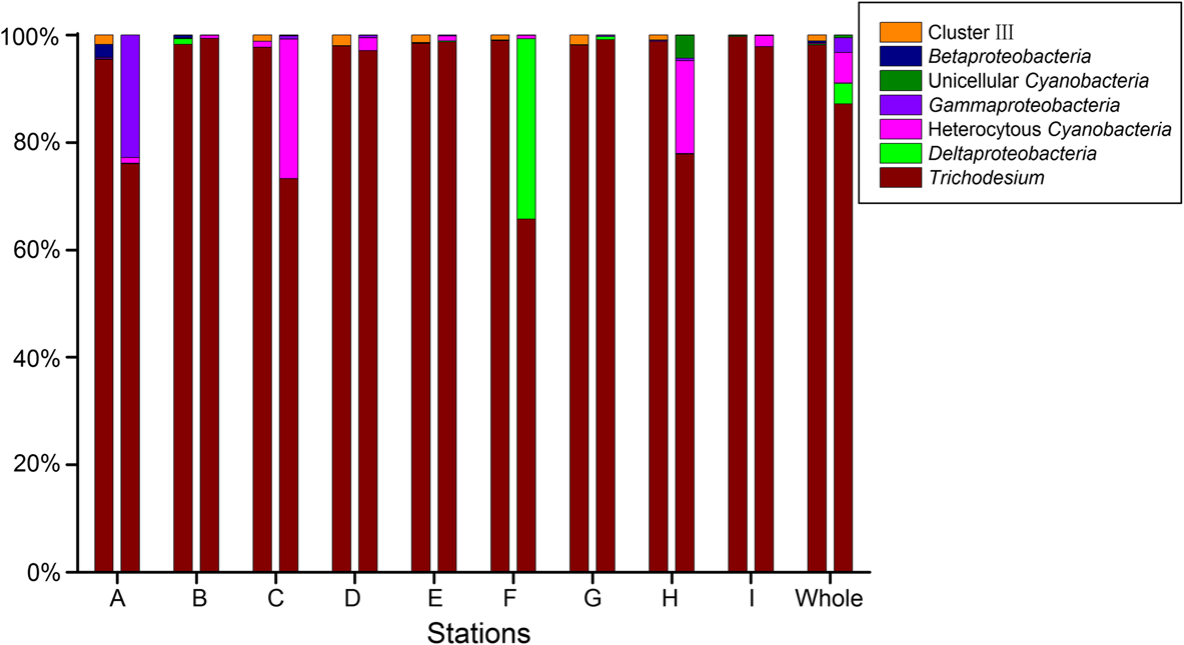
Composition of *nifH* taxa based on DNA- and RNA-derived libraries. For all stations (A–I), the left bars represent the DNA-derived libraries and the right bars the RNA-derived libraries; “whole” refers to the average relative abundance of all samples

*Trichodesmium* predominated in both the total and the active fractions of the PADs communities across all stations, representing, on average, 98% and 87% of the *nifH* DNA and *nifH* RNA sequences, respectively (Fig. 4). Eight of the 20 most abundant OTUs were affiliated with *Trichodesmium*, thus indicating a divergent phylogenesis of the *Trichodesmium* nitrogenase gene (Fig. 3). Specifically, the relative abundance of *Trichodesmium* was relatively stable at all stations, ranging from 95.5 to 99.7% within the individual DNA libraries (Fig. 4). However, the variation in the relative abundance of this genus was more pronounced in the RNA libraries, ranging from 65.7% at station F to 99.4% at station B (Fig. 4).

Some diazotrophs were enriched in the RNA libraries compared to their relative abundance in the DNA libraries, while for others the opposite was the case (Fig. 4). For example, *Richelia*-like heterocystous cyanobacteria were absent or of lower abundance (0.2%) in the total PADs communities but reached moderate abundances in eight of the nine stations (Fig. 4). Plankton-associated *Deltaproteobacteria* were over-represented in the active PADs community of station F, and a similar pattern was observed for *Gammaproteobacteria* at station A (Fig. 4). Unicellular cyanobacteria, whose *nifH* sequences clustered together with those of *Crocosphaera watsonii* WH 8501 (Fig. 3), accounted for only 0.01% of the *nifH* DNA sequences, but their contribution to the *nifH* RNA sequences was higher (0.5%). Conversely, taxa affiliated with cluster III and *Betaproteobacteria* accounted for an average of 1.11% and 0.33%, respectively, of the abundance based on the DNA libraries across stations, but their abundances were negligible in the RNA libraries (Fig. 4).

We then explored the relationship between community composition and the corresponding environmental variables. Since the physical and chemical parameters of the water column vary with water depth and sampling site (see Additional file 1: Table S3 and Figure S1), separate RDA analyses were performed for surface and averaged (across depths) environmental parameters. Total and active PADs communities significantly correlated with sea surface pH, water column average pH, concentrations of average nitrate plus nitrite, and the average ammonium concentration (Additional file 1: Table S4). Several environmental factors significantly influenced the abundance and activity of *Trichodesmium*. Both total and active *Trichodesmium* highly correlated with sea surface pH (*P* = 0.019), water column average pH (*P* = 0.021), and marginally correlated with average nitrate plus nitrite concentrations (*P* = 0.100) (Additional file 1: Figure S3 and Table S4). However, there was no significant correlation between total and/or active heterocystous cyanobacteria and any of the environmental variables (Additional file 1: Figure S3).

### Proportions of abundant OTUs

**Fig. 5:**
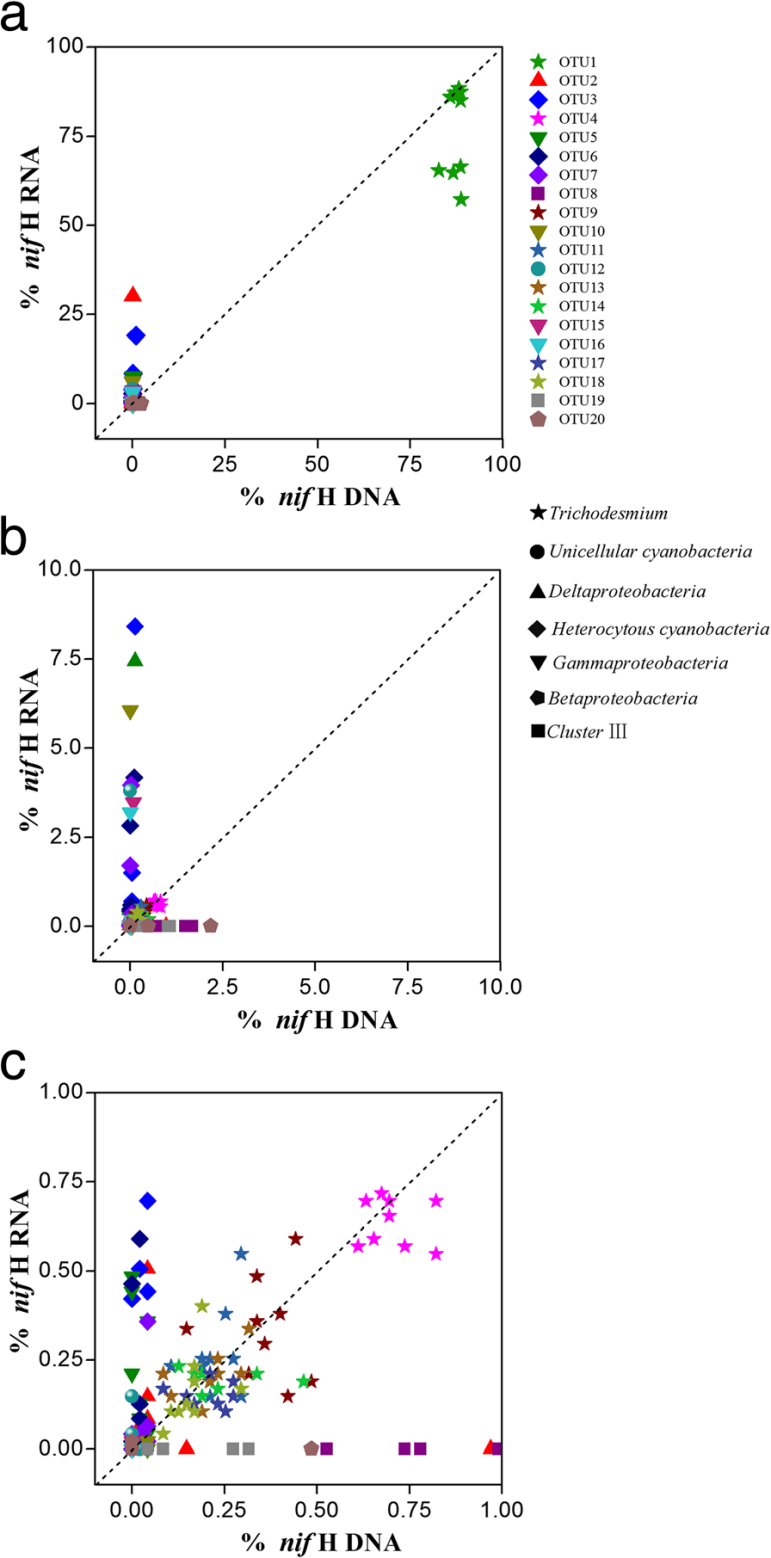
Relationship between *nifH* RNA and *nifH* DNA frequencies of the dominant OTUs. The panels show all the 20 most abundant OTUs (**a**) as well as abundant OTUs accounting for < 10% (**b**) and < 1% (**c**) of the abundance in both the DNA and RNA libraries. The dotted line indicates a 1:1 ratio. A deviation from the 1:1 line is expected for OTUs with a notably higher proportion in either the DNA or the RNA library, resulting in a distribution of the dots towards the *x* or *y* axis

To assess whether the 20 most abundant OTUs accounted for a disproportionate amount of *nifH* gene activity, we compared the relative abundances (frequencies) of each OTU in the DNA and RNA libraries (Fig. 5). The relative abundance of OTU1 was > 50% in both libraries in all samples, while the remaining 19 OTUs were less abundant (Fig. 5). The frequencies of all eight OTUs affiliated with *Trichodesmium* were approximately equal in the DNA and RNA libraries, with disproportionate frequencies determined for the remaining 12 OTUs. For example, OTUs affiliated with members of heterocystous cyanobacteria, unicellular cyanobacteria, *Gammaproteobacteria*, and *Deltaproteobacteria* were of relatively high but varying frequencies in the *nifH* RNA libraries (Fig. 5) whereas the OTUs of members of the *Betaproteobacteria* and diazotrophs of cluster III were of negligible frequency in the RNA but not the DNA libraries (Fig. 5).

### *NifH* gene expression level

**Fig. 6:**
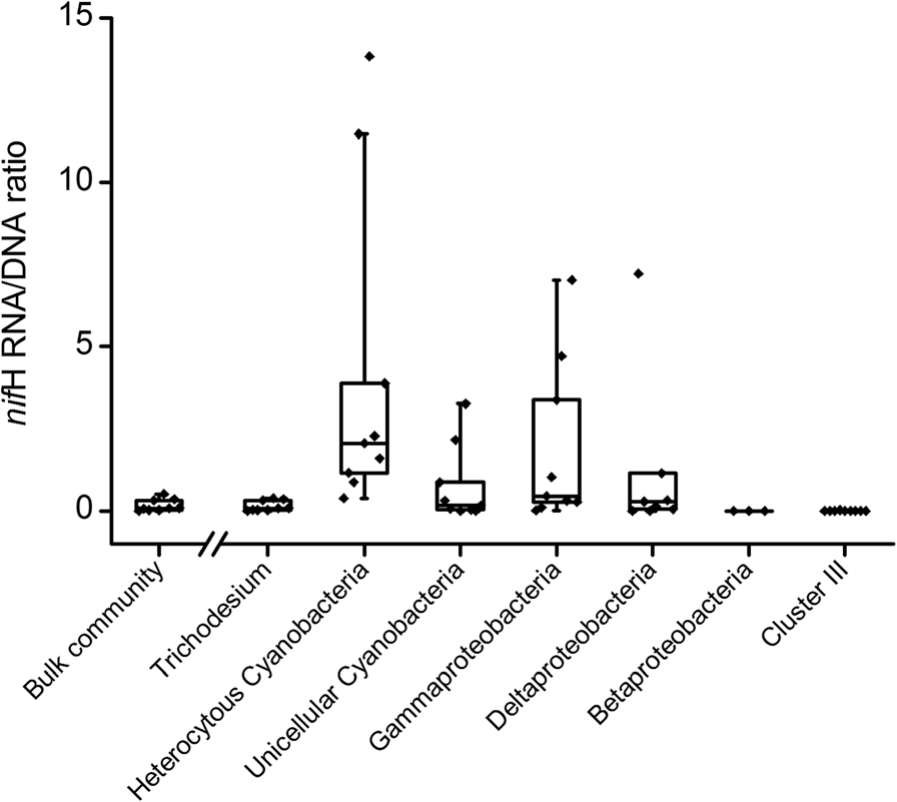
The *nifH* mRNA expression level of the main groups. The expression ratio is based on the quantitative PCR results of each sample combined with the average taxonomic frequencies in the *nifH* DNA and RNA libraries. The dots represent the *nifH* RNA/DNA ratio of each sample. For some samples, the ratio is not provided because no sequence was found in the DNA libraries

The *nifH* gene expression level of the bulk PADs communities ranged from 0.0042 to 0.52, with an average *nifH* RNA/DNA ratio of 0.17 (Fig. 6). To further quantify *nifH* gene expression by the different PADs groups, we calculated the *nifH* RNA/DNA ratio of the main groups by combining the bulk community *nifH* RNA/DNA ratio with the frequencies of the dominant groups. Heterocystous cyanobacteria were the most active group, based on *nifH* RNA/DNA ratios ranging from 0.39 to 13.82 (average *nifH* RNA/DNA ratio 3.51). *Trichodesmium* predominated in the total and active PADs communities (Fig. 4), but *nifH* gene expression by this genus (average *nifH* RNA/DNA ratio 0.15) was somewhat lower than that of the bulk community (0.17). Unicellular cyanobacteria, *Deltaproteobacteria* and *Gammaproteobacteria* were present at low abundances (Fig. 4) but exhibited high-level *nifH* expression (Fig. 6), whereas *nifH* mRNA expression by diazotrophs of cluster III and *Betaproteobacteria* was nearly undetectable*.* Due to high between-sample variations, which might be caused by environmental differences, there was no statistically significant difference in the *nifH* RNA/DNA ratio of the analyzed PADs groups.

## Discussion

The aim of this study was to elucidate the community structure and *nifH* mRNA expression of diazotrophs associated with plankton with a particle size > 100 μm in northern South China Sea. Our results showed that the active PADs communities were taxonomically distinct from the total communities. *Trichodesmium* predominated among the PADs of the euphotic zone, with approximately equal abundances in the DNA and RNA libraries. Differences in the *nifH* gene expression levels of the different phylogenetic groups of diazotrophs may be related to the life forms and symbiotic strategies of these organisms.

### Active communities are more diverse and divergent than total communities

In determinations of the microbial communities in a particular environment, higher community diversity in DNA- than in RNA-derived libraries is typical and has been demonstrated in several 16S rRNA-based studies [33, 36–39]. Here we directly tracked *nifH*-defined communities, because *nifH* gene may provide better insight into the diversity and composition of the communities that are capable of nitrogen fixation in an environment than those extrapolated from 16S rRNA data. However, our *nifH* sequence analysis revealed a higher alpha-diversity in the active (based on RNA) than in the total (based on DNA) communities of PADs (Fig. 1). The evenness of the total PADs communities was also lower than that of the metabolically active communities, as indicated by the very small number of highly dominant taxa. The *nifH* gene-based OTU abundances from the DNA analysis do not always predict which diazotrophic OTU is actively expressing *nifH* assessed in the RNA analysis. The former one is presumably considered a proxy for the dominant diazotrophs in a given sample, whereas the latter offers a representation of metabolically active diazotrophs with regard to nitrogen fixation processes. A high alpha diversity in a given community could be a result of high evenness. As such, a higher evenness obtained from *nifH* RNA-based OTU analysis accounts for closer number among individuals that were actively expressing *nifH*, relative to the communities comprising a few highly dominant diazotrophs detected in *nifH* DNA based approach. This is supported by the prevalence of *Trichodesium* in all DNA-derived communities and the decline in its abundance in the RNA-derived communities, whereas some taxa that were rare in the total communities were of moderate abundance in the active communities (Fig. 4). Thus, although the low-abundance groups represented a small proportion of the total PADs community biomass, they contributed a disproportionately high amount of the activity, presumably due to active transcription of nitrogenase mRNA under suitable growth conditions.

Previous studies have confirmed the dissimilarities between total and active diazotrophic communities [40, 41], but the variability within communities of total and active diazotrophshas not been quantitatively investigated. Given that changes occur faster in RNA than in DNA [42], we hypothesized a more pronounced variability in the active diazotrophic communities (RNA) across stations than in the total communities (DNA). Indeed, variations in the community structures of PADs among sampling sites were observed and were independent of the presence or activity of the cells. However, in accordance with our hypothesis, this variation was significantly higher in the active fraction of the diazotrophic communities than in the total communities (Fig. 2b). Moreover, the differences between the active communities at different sites were mainly caused by the replacement of taxa, rather than changes in the abundance of common taxa (Fig. 4), suggesting that different selective pressures determine the turnover in membership of metabolically active diazotrophs in the studied ecosystems. Accordingly, active diazotrophic assemblages may better reflect the response of PADs to environmental alterations.

### A varying level of metabolic activity among rare taxa

Given that some taxa tend be to conditionally rare [43], investigations of membership turnover in rare but active populations of natural communities may shed light on microbial diversity and succession. In our study, rare but active PADs groups included heterocystous cyanobacteria, unicellular cyanobacteria, *Deltaproteobacteria* and *Gammaproteobacteria* (Figs. 4 and 6). Members of the heterocystous cyanobacterial genus *Richelia* were the most transcriptionally active group. The heterocystous cyanobacteria *Richelia* has been extensively reported as a diatom symbiote, including the investigations in the South China Sea [34, 35], and was identified to actively transfer nitrogen to their diatom hosts [15]. The association of diazotrophic unicellular cyanobacteria with phytoplankton has been the subject of recent studies [11, 27, 44], including a report describing a high nitrogen fixation rate and rapid nitrogen transfer from symbiotic unicellular cyanobacteria to their hosts, evidenced by N-isotope-based nano-SIMS technology [11]. The highly active transcription of nitrogenase mRNA by intracellular symbiotic *Richelia* and unicellular cyanobacteria detected in our study is in agreement with previous studies reporting high nitrogen fixation rates by those diazotrophs [11, 15]. Intracellular symbiotic strategies and nutrient exchange mechanisms may facilitate a high efficiency of nitrogen fixation by *Richelia* as well as unicellular cyanobacteria.

High nitrogenase mRNA transcription level was also detected in *Deltaproteobacteria* and *Gammaproteobacteria*, previously reported to be dominant diazotrophic groups [35, 45]. In our study, although *Deltaproteobacteria* was present at low abundance, its level of *nifH* mRNA transcription activity was high. This group was detected in copepods in previous studies [24, 26] whereas in the case of diazotrophic *Gammaproteobacteria* little is known about its symbiotic relationships. Fluorescence in situ hybridization studies of diazotrophic *Gammaproteobacteria* may lead to the identification of its host and thus provide insights into the ecological importance of this PADs group.

### The inactive diazotrophic groups

Nitrogenase is a multi-component enzyme with several different forms, although the *nifH* gene encodes a highly conserved subunit [30]. However, the *nifH* gene also shares a similarity with *nifH*-like genes in non-diazotrophic organisms, such that many *nifH* sequences retrieved from environmental clones are in fact pseudogenes [46, 47]. In this study, *nifH* sequences belonging to cluster III were the second most abundant group in the *nifH* DNA libraries, but few transcripts were detected in the *nifH* RNA libraries for all stations (Figs. 4 and 5). Considering that the cluster III *nifH* sequences obtained in this study were not transcribed, whether they were pseudogenes or indeed belonged to inactive groups was unclear. The reference sequences that clustered closely with the sequences of cluster III (Fig. 3) originated from zooplankton and the termite gut [24, 48]. In addition, the nitrogenase activity of PADs was shown to be much higher in starved copepods than in full-gut copepods [26]. It was therefore hypothesized that, the presence of sufficient bio-available nitrogen in the zooplankton gut down-regulates nitrogenase gene expression [26]. Similarly, the cluster III diazotrophs found in our study might be those inactive members associated with zooplankton, which would imply that zooplankton-associated heterotrophic diazotrophs are suppliers of extra nitrogen for their hosts, especially under conditions of starvation.

### *Trichodesmium* dominate the PADs

The results of microscopy-based cell counting showed a relatively lower *Trichodesmium* abundance (1.29 × 10^3^–8.22 × 10^3^ trichomes m^− 2^ in the euphotic waters, Additional file 1: Figure S2) in the South China Sea than previously reported [34, 35, 49]. The differences may have been due to the use of different sampling methods, as the net-tow (100-μm mesh) collection of plankton in our study would have excluded free-living or smaller aggregates of *Trichodesmium* cells, both of which are captured in previous studies using smaller pore-size filters (0.2-, 10- or 20-μm) [34, 35, 49]. Nevertheless, the sampling method used in our study was chosen to ensure collection of the PADs fraction while excluding free-living diazotrophs, as that different patterns for PADs and whole diazotrophic communities were expected.

*Trichodesmium* is one of the dominant diazotrophs in tropical and subtropical oceans [5, 6, 50]. In the South China Sea, heterotrophic *Proteobacteria* was shown to dominate the diazotrophic communities [34, 35]. However, consistent with the results of Farnelid and colleagues, who reported the predominance and enrichment of cyanobacteria, including *Trichodesmium*, in diazotrophic communities associated with sinking particles [27], we found that *Trichodesmium* accounted for nearly all of the PADs (~ 98%) in our samples. It seems that autotrophic cyanobacterial diazotrophs such as *Trichodesmium* tend to dominate in large particle-size fraction of the diazotrophic community inhabiting the euphotic-zone, while heterotrophic diazotrophs tend to be free-living. Alternatively, the disproportional enrichment of *Trichodesmium* in the > 100 μm size fraction could be resulted from colonization of their cells. Thus, our collection method revealed a difference in the community structure of bulk diazotrophs vs. PADs associated with large particles.

We also found that *Trichodesmium* accounted for the majority (87%) of the nitrogenase mRNA transcripts of PADs (Fig. 4). However, an analysis of the *nifH* RNA/DNA ratio showed that the *nifH* gene expression level of *Trichodesmium* was lower than that of rare but active groups (e.g., heterocystous cyanobacteria) (Fig. 6). In a previous isotope-based investigation, more than half of the *Trichodesmium* cells were incapable of fixing nitrogen [11]. Therefore, changes in the abundance of diazotrophic groups, determined using *nifH* DNA sequences, may not reflect changes in the nitrogen fixation activity of the respective members, whose ability to fix nitrogen varies. However, newly developed isotope techniques, especially NanoSIMS, allow the nitrogen fixation rate of specific groups to be investigated at the single cell level [11, 51].

## Conclusions

In this study, the total and active communities of PADs in the northern South China Sea were inventoried, and the *nifH* gene expression level by different PADs groups were estimated. Active communities of PADs were significantly more divergent than the total communities. Overall, however, the PADs communities were taxonomically diverse, with different phylogenetic groups differing in their *nifH* mRNA transcription activity. Despite the low abundance of heterocystous cyanobacteria, unicellular cyanobacteria, *Deltaproteobacteria*, and *Gammaproteobacteria*, the *nifH* transcriptional activity of these groups was high, whereas *Trichodesmium*was highly abundant but its transcriptional activity was moderate, and *Betaproteobacteria* and diazotrophs of cluster III were of high abundance but of low *nifH* transcriptional activity. Our results extend those of previous studies by specifically characterizing the distribution, abundance, and transcriptional activity of PADs in the northern South China Sea.

Nonetheless, much remains to be learned about the diversity and structure of PADs communities, including the symbiotic relationships between the various species of PADs and their hosts. Determinations of the amount of nitrogen fixation contributed by each PADs group and its impact on both host metabolism and the functioning of marine ecosystems are needed as well. Manual sorting of plankton prior to amplification and sequencing is a feasible way to assess the specificity of the symbiotic relationships between PADs and host plankton species [25, 26]. The sequence information obtained in our study will aid in designing nucleotide probes for the in-situ visualization of specific diazotrophs in their plankton hosts. Furthermore, our findings highlight the importance of low-abundance PADs community members in understanding nitrogen fixation in marine ecosystems, but also the need for more detailed insights into the symbiotic strategies and contribution to nitrogen fixation of diazotrophic communities.

## Methods

### Study sites and sample collection

**Fig. 7:**
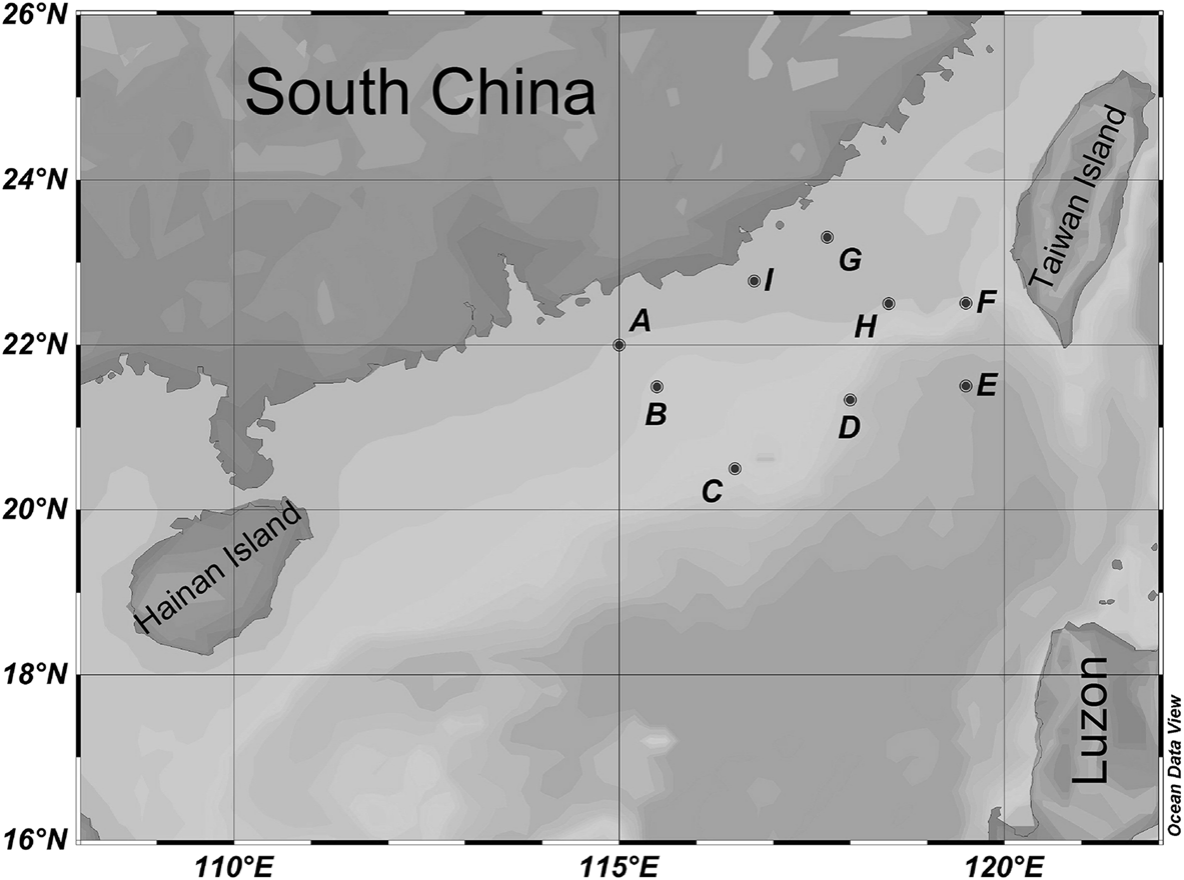
Location of the sampling stations. The map was generated using ODV software (Schlitzer, R., Ocean Data View, http://odv.awi.de, 2015)

Seawater used for this study was collected on *R/V Shiyan3* cruise WPOS (20 October–10 November 2014) to the northern South China Sea. Plankton samples were obtained at nine sites using a vertical haul with a 100-μm-mesh, 0.4-m-diameter plankton net (Fig. 7; see Additional file 1: Table S1 for more information on the sampling locations). The samples were rinsed with 0.22-μm-filtered seawater and the final volume was adjusted to 1 L. The collected samples were thoroughly mixed and a 250-ml aliquot was fixed with 0.1% Lugol’s iodine solution for *Trichodesmium* counting by microscopy [52]. The remaining volume of each sample was incubated in DNA/RNA Sample Protector Buffer (Takara BioInc. Japan), and stored at − 20 °C until nucleic acid extraction. Additionally, seawater samples were collected using a CTD system (Sea-Bird Scientific Inc. WA, USA) fitted with temperature, conductivity, pH and dissolved oxygen probes. The seawater was filtered through a 0.22-μm PEGF membrane (Millipore, Darmstadt, Germany) and then stored at − 20 °C for nutrient concentration measurements [53].

### Nucleic acid extraction, cDNA synthesis and Illunima sequencing

By centrifuging at 5000×g for 5 min, the supernatant of DNA/RNA protector buffer was removed from each sample prior to nucleic acid extraction. DNA and RNA were simultaneously extracted using a total DNA/RNA isolation kit (Omega Bio-tek, Inc. GA, USA) and their concentrations were measured using a NanoDrop 2000 spectrophotometer (Thermo Fisher Scientific Inc. DE, USA). Genomic DNA was removed from the RNA extracts by DNase digestion (Takara BioInc. Japan) prior to cDNA synthesis using a PrimeScript RT reagent kit (Takara BioInc. Japan). The 20-μl reverse transcription reaction contained 2 μl (< 2 μg) of RNA. A negative control without reverse transcriptase was included to check for genomic DNA contamination of the cDNA.

The *nifH* gene of the DNA and cDNA were amplified using the primer pair PolF and PolR [54] combined with barcode sequences. Amplification was performed in reaction mixtures consisted of 25 μl Ex Taq (2×) (Takara BioInc. Japan), 1 μl of primer PolF (10 μM), 1 μl of primer PolR (10 μM) and 1 μl of DNA/cDNA in a 50-μl final volume. The thermal profile was as follows: initial denaturation at 94 °C for 5 min, followed by 35 cycles of denaturation at 94 °C for 30 s, annealing at 54 °C for 45 s and extension at 72 °C for 45 s, and final elongation at 72 °C for 10 min. Libraries were constructed from the purified PCR products of each sample. The 2 × 250-bp paired-ends sequencing was conducted on a HiSeq 2500 platform, Illumina (Genewiz, Suzhou, China).

### Phylogenetic analysis

Quality trimming was done using Btrim with a quality score threshold to 20 [55]. Forward and reverse reads were merged using FLASH [56]; the overlaps of the merged reads more than 30 bp and mismatch less than 5% were kept, otherwise filtered out. Sequences with a length < 300 base pairs and ambiguous bases > 0 were removed. The forward and reverse primers were trimmed before further analysis. Operational taxonomic units (OTUs) were classified using UCLUST, based on 94% sequence similarity, as described previously [57]. Chimeric OTUs were removed using UCHIME in de novo mode [58]. Frame-shift errors in the sequences were checked and corrected using RDP FrameBot [59]. All libraries were subsampled with 100 iterations to 4746 sequence reads (smallest library size) to standardize uneven sequencing depths, resulting in a normalized OTUs table.

The validated *nifH* sequences were translated into amino acid sequences for phylogenetic analysis. Among the OTUs in the *nifH* DNA and RNA libraries, whose normalized reads in all samples were aggregated, the 20 most abundant OTUs across samples were assigned by ranking the aggregated reads. Representative sequences of the 20 most abundant OTUs (accounting for 90.3% of the total sequences) were checked against the closest related sequences in the GenBank database and in an ARB *nifH* database [60]. We selected the reference sequences based on the nucleotide sequence similarity and the score obtained from the BLAST: 1) sequences from the top 3–5 matching hits and 2) one sequence derived from pure culture isolates that scored highest. Based on these criteria, a total of 39 sequences were retrieved, where 26 sequences were derived from environmental clones, and the other 13 sequences from pure culture isolates. The *nifH* protein sequences of the 20 most abundant OTUs and 39 reference sequences were used to construct the phylogenetic tree. A neighbor-joining phylogenetic tree was constructed using MEGA software (v6.02) [61]. Bootstrap confidence values of the nodes were obtained by 1000 iterations. Substitution model of Poisson, uniform rates among sites and homogeneous pattern among lineages were used for MEGA. The taxonomic affiliation of the 20 abundant OTUs was assigned based on their phylogenetic relatedness to the reference sequences on the tree. Representative *nifH* gene sequences were deposited in the GenBank database under accession numbers KY774953–KY774972.

### Quantification of *nifH* mRNA expression level

The primers PolF and PolR [54] were used to quantify the ratio of *nifH* mRNA/*nifH* DNA. The amplification was performed in 20-μl reaction mixtures containing 10 μl of SYBR Premix Ex TaqII (2×) (Takara BioInc. Japan), 0.8 μl of primer PolF (10 μM), 0.8 μl of primer PolR (10 μM) and 1 μl of DNA/cDNA sample. Quantitative PCR was carried out using the Light Cycler 480 System (Roche Molecular Systems, Inc. CA, USA) and the following PCR conditions: 2 min at 95 °C, 45 cycles of 94 °C for 30 s, 54 °C for 45 s, and 72 °C for 45 s. All reactions were performed in triplicate. The specificity of the amplification was confirmed by melting-curve analysis and electrophoresis in a 2% agarose gel.

The ratio of *nifH* mRNA and *nifH* DNA in the bulk communities was calculated according to Eq. 1:

1 $$ {R}_{bulk}={E}^{\varDelta Ct} $$

where *R* is the resulting ratio of *nifH* mRNA/*nifH* DNA, *E* is the amplification efficiency, and *ΔCt* the cycle threshold difference between *nifH* mRNA and *nifH* DNA. The amplification efficiency was calculated using established standards, as described in [62]. The *nifH* RNA/DNA ratio of the main PADs groups was further calculated by multiplying the *nifH* RNA/DNA ratio obtained from the bulk community by the respective frequencies of the dominant groups (Eq. 2):

2 $$ {R}_{\mathrm{group}}={R}_{\mathrm{bulk}}\left({f}_{\mathrm{RNA}}/{f}_{\mathrm{DNA}}\right) $$

where *f* is the frequency of each group in either the RNA or the DNA library.

### Statistical analysis

Diversity indexes were calculated based on 100 resampled OTUs tables. The Chao1 index, Shannon index (H), Simpson index (D), and Pielou evenness (J) were calculated to determine the alpha-diversity of the *nifH*-derived communities. Student’s t-test was used to identify the significance of the difference in the diversity indexes between the RNA and DNA libraries. A Monte Carlo permutation test was performed to evaluate the correlation between environmental attributes and *nifH* sequences. Differences in community composition among the nine sampling stations and between the total and active PADs communities were identified using non-metric multidimensional scaling (NMDS) with a Bray-Curtis dissimilarity distance. An analysis of beta-dispersion [63] was used to quantify between-community variations in the total and the active PADs communities, and a redundancy analysis (RDA) to explore the relationship between diazotrophic groups and environmental parameters. Statistical analyses in this study were performed using the R vegan package (v3.5.1) and an R-based pipeline, as previously described [62].

## Additional file

Additional file 1: **Figure S1.** Water quality profiles at each station as determined by the CTD sampler. **Figure S2.** Abundance of Trichodesmium in at the nine stations. Cell numbers were determined by microscopic counting. **Figure S3.** Redundancy analysis (RDA) ordination plot showing the relationships between environmental variables and taxa in the DNA and RNA libraries. **Table S1.** Geolocation of the stations, sampling depth, and sample volume. **Table S2.** Alpha-diversity indexes of the total and active communities in each sample. **Table S3.** Nutrient concentrations at the sampling stations. **Table S4.** Monte Carlo permutation test of the effects of environmental variables on the *nifH*-derived communities. **Table S5.** The C_T_ values obtained from the technical triplicate of quantitative PCR. (PDF 899 kb)

## Data Availability

The datasets used and/or analyzed for the present study are available from the corresponding author on reasonable request. The nucleotide sequences have been deposited in GenBank nucleotide database of NCBI under accession numbers (KY774953–KY774972).
