# Supplementary material for: Analysis of nifH DNA and RNA reveals a disproportionate contribution to nitrogenase activities by rare plankton-associated diazotrophs
Source: BMC Microbiol. 2019 Aug 15;19:188. doi: 10.1186/s12866-019-1565-9 (PMC6694519; doi:10.1186/s12866-019-1565-9)
Supplement: Supplementary file 1 — Figure S1. Water quality profiles at each station as determined by the CTD sampler. Figure S2. Abundance of Trichodesmium in at the nine stations. Cell numbers were determined by microscopic counting. Figure S3. Redundancy analysis (RDA) ordination plot showing the relationships between environmental variables and taxa in the DNA and RNA libraries. Table S1. Geolocation of the stations, sampling depth, and sample volume. Table S2. Alpha-diversity indexes of the total and active communities in each sample. Table S3. Nutrient concentrations at the sampling stations. Table S4. Monte Carlo permutation test of the effects of environmental variables on the nifH-derived communities. Table S5. The CT values obtained from the technical triplicate of quantitative PCR. (PDF 899 kb) [file 12866_2019_1565_MOESM1_ESM.pdf]

Supplementary Material for

Analysis of *nifH* DNA and RNA reveals a disproportionate contribution to nitrogenase activities by rare plankton-associated diazotrophs

Qingsong Yang[1, 3],Junde Dong[1, 2], Manzoor Ahmad[1, 3], Juan Ling[1], Weiguo Zhou[1, 3], Yehui Tan[1], Yuanzhou Zhang[4], Dandan Shen[5]*, Yanying Zhang [1, 2]*

[1]CAS Key Laboratory of Tropical Marine Bio-resources and Ecology, Guangdong Provincial Key Laboratory of Applied Marine Biology, South China Sea Institute of Oceanology, Chinese Academy of Sciences, 510301 Guangzhou, China

[2]Tropical Marine Biological Research Station in Hainan, South China Sea Institute of Oceanology, Chinese Academy of Sciences, 572000 Sanya, China

[3]University of Chinese Academy of Sciences, 100049 Beijing, China

[4]State Oceanic Administration Sansha Marine Environmental Monitoring Center Station, 570311, Haikou, China

[5]Section of Biological Oceanography, Leibniz Institute for Baltic Sea Research, 18119, Warnemünde, Germany

***Correspondence:**

Dandan Shen, dand.shen@gmail.com

Yanying Zhang, zyy@scsio.ac.cn

Contents:

Supplementary Fig. S1

Supplementary Fig. S2

Supplementary Fig. S3

Supplementary Table S1

Supplementary Table S2

Supplementary Table S3

Supplementary Table S4

Supplementary Table S5

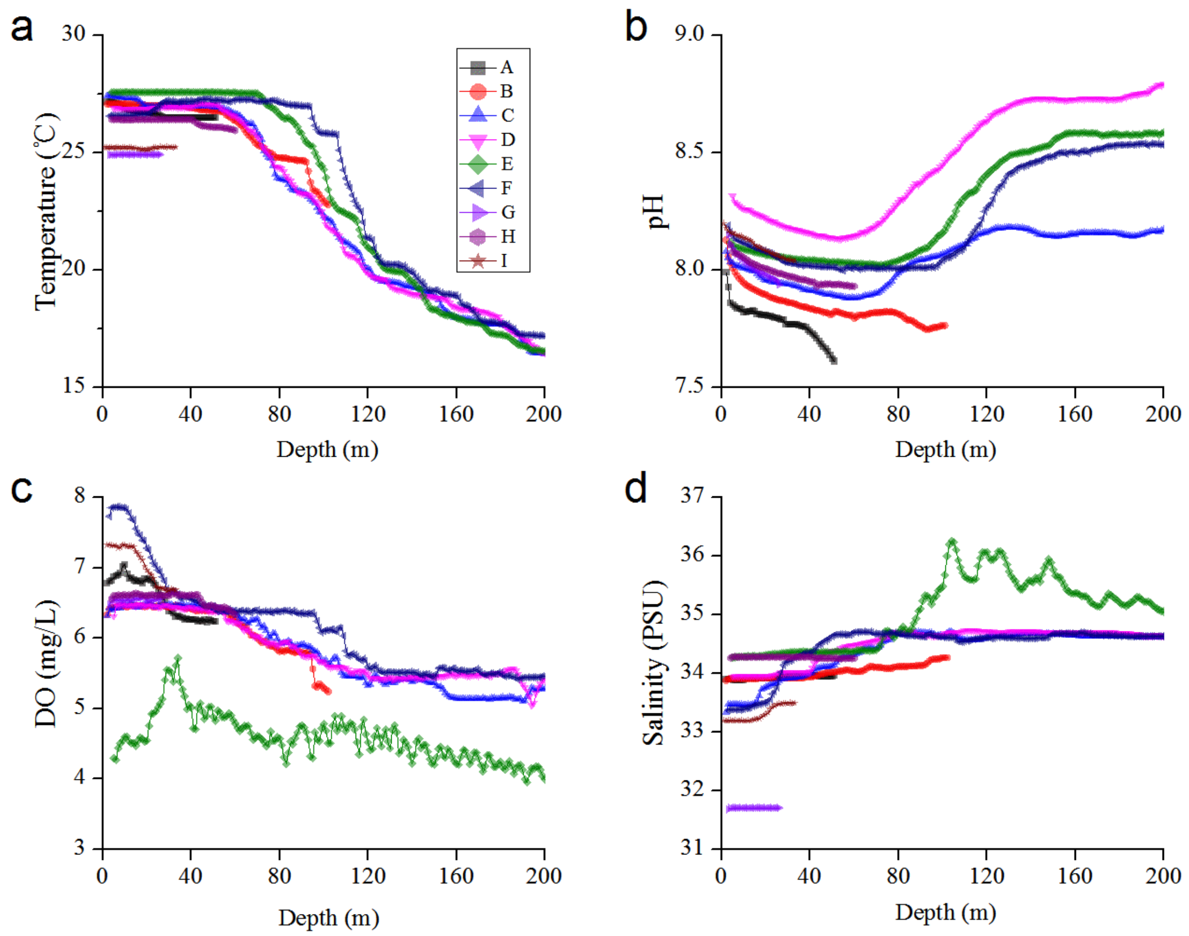

Fig. S1 Water quality profiles at each station as determined by the CTD sampler.

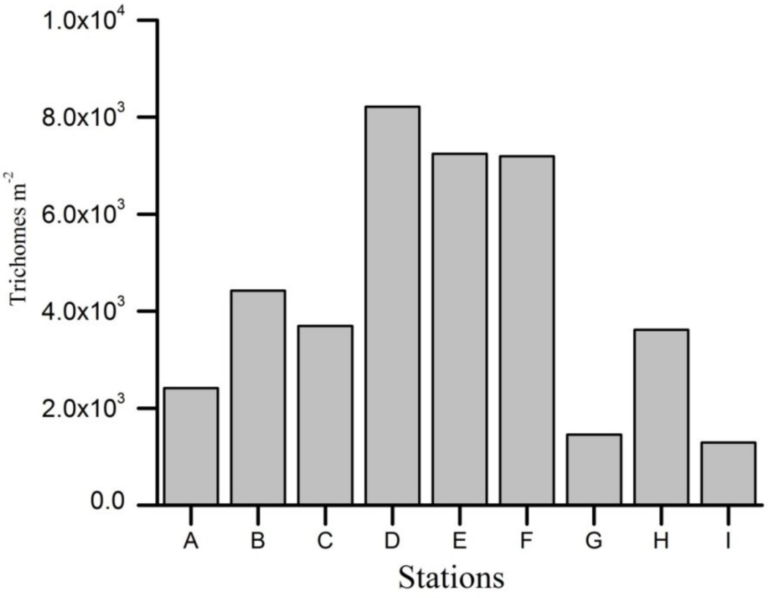

Fig. S2 Abundance of *Trichodesmium* in at the nine stations. Cell numbers were determined by microscopic counting.

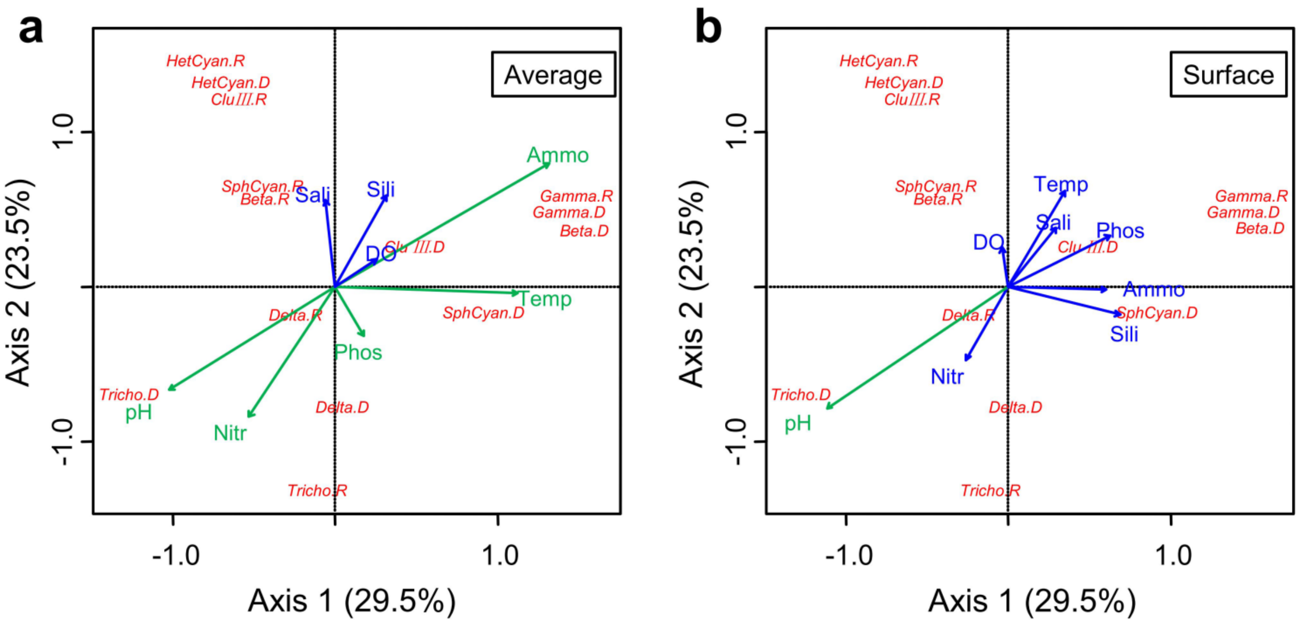

Fig. S3 Redundancy analysis (RDA) ordination plot showing the relationships between environmental variables and taxa in the DNA and RNA libraries: (a) average values from the water column and (b) the values from the sea surface. Taxa are shown in red (R: taxa derived from the RNA library; D: taxa derived from the DNA library). Tricho, *Trichodesmium*; HetCyan, heterocystous cyanobacteria; SphCyan, spherical cyanobacteria; Gamma, *Gammaproteobacteria*; Delta, *Deltaproteobacteria*; Beta, *Betaproteobacteria*; Clu III, cluster III. Environmental variables that correlated significantly with the taxa are indicated in green, otherwise in blue. Temp, temperature; DO, dissolved oxygen; Sali, salinity; Nitr, nitrate+nitrite; Phos, phosphate; Ammo, ammonium; Sili, silicate.

Table S1 Geolocation of the stations, sampling depth, and sample volume

**Station**	**Geolocation**	**Water depth (m)**	**Sampling depth (m)**	**Sample volume (L)**
A	21°59.789′, 114°59.924′	57.8	55	12304.9
B	21°29.443′, 115°29.513′	108.7	105	22549.1
C	20°30.026′, 116°30.022′	435.5	200	18840.0
D	21°19.931′, 118°0.070′	1475.8	200	41860.1
E	21°30.040′, 119°29.665′	2999.0	200	36914.6
F	22°30.142′, 119°30.078′	227.0	200	36679.1
G	23°18.133′, 117°42.110′	33.9	31	7418.3
H	22°29.836′, 118°30.106′	64.3	60	18427.9
I	22°46.199′, 116°44.916′	33.3	31	6594.0

Table S2 Alpha-diversity indexes of the total and active communities in each sample

	Station	Shannon index	Simpson index	Pielou evenness	Chao1
Total communities	A	1.21	1.45	0.23	223.02
	B	0.97	1.32	0.19	200.25
	C	1.00	1.33	0.20	200.55
	D	0.86	1.27	0.17	183.77
	E	0.89	1.28	0.18	189.52
	F	0.89	1.28	0.18	187.39
	G	0.91	1.30	0.18	217.62
	H	0.86	1.27	0.17	196.36
	I	1.03	1.33	0.20	200.64
Active communities	A	1.84	2.31	0.35	247.45
	B	0.99	1.33	0.20	192.10
	C	1.55	2.20	0.30	211.91
	D	1.09	1.38	0.21	227.56
	E	1.00	1.32	0.19	214.92
	F	1.56	2.39	0.29	268.32
	G	0.92	1.29	0.18	205.18
	H	1.80	2.21	0.34	264.03
	I	1.07	1.38	0.21	200.90

Table S3 Nutrient concentrations at the sampling stations.

Station	Column average(μmol L^{-1})				Surface water(μmol L^{-1})			
	Nitrite+ Nitrate	Ammonium	Phosphate	Silicate	Nitrite+ Nitrate	Ammonium	Phosphate	Silicate
A	0.39	0.57	0.27	5.00	0.19	0.44	0.27	3.95
B	0.85	0.14	0.20	4.00	0.30	0.48	0.07	1.94
C	1.79	0.18	0.32	6.32	0.32	0.43	0.12	1.65
D	2.37	0.13	0.42	5.22	0.22	0.00	0.03	2.13
E	3.22	0.04	0.47	4.69	0.08	0.62	0.07	1.56
F	1.45	0.08	0.21	3.69	0.75	0.08	0.12	2.23
G	4.93	0.00	0.38	4.92	5.38	0.00	0.37	5.78
H	0.37	0.28	0.12	1.97	0.63	0.00	0.17	1.56
I	0.84	0.48	0.09	0.93	0.81	0.5.	0.07	0.98

Table S4 Monte Carlo permutation test of the effects of environmental variables on the *nifH*-derived communities

	Water column (average)	Sea surface
Nitrate+Nitrite	0.100*	0.503
Ammonium	0.064*	0.402
Phosphate	0.094*	0.608
Silicate	0.405	0.969
Temperature	0.040**	0.732
Salinity	0.910	0.608
pH	0.021**	0.019**
Dissolved oxygen	0.990	0.290

Asterisks indicate the level of significance: *$P < 0.1$; **$P < 0.05$

Table S5 The C_T values obtained from the technical triplicate of quantitative PCR.

Sample name	qPCR C_T value				
	Replicate 1	Replicate 2	Replicate 3	Mean	Standard Error
nifH_DNA_A	26.45	26.21	26.29	26.32	0.12
nifH_DNA_B	27.45	27.60	27.04	27.36	0.29
nifH_DNA_C	27.04	27.06	27.16	27.09	0.06
nifH_DNA_D	22.19	22.67	22.22	22.36	0.27
nifH_DNA_E	22.34	22.23	22.11	22.23	0.11
nifH_DNA_F	24.44	24.05	24.02	24.17	0.23
nifH_DNA_G	24.66	25.08	24.54	24.76	0.28
nifH_DNA_H	23.46	23.63	23.44	23.51	0.10
nifH_DNA_I	25.53	25.64	25.67	25.61	0.074
nifH_RNA_A	33.20	33.65	33.67	33.51	0.26
nifH_RNA_B	31.21	31.22	31.52	31.32	0.18
nifH_RNA_C	30.46	30.56	30.01	30.34	0.29
nifH_RNA_D	29.75	29.75	29.91	29.80	0.09
nifH_RNA_E	28.10	28.3	28.2	28.20	0.10
nifH_RNA_F	30.32	30.24	30.25	30.27	0.04
nifH_RNA_G	28.51	28.49	28.64	28.55	0.08
nifH_RNA_H	33.35	33.68	34.18	33.74	0.42
nifH_RNA_I	31.60	31.12	31.10	31.27	0.28
